# Sparse representation approaches for the classification of high-dimensional biological data

**DOI:** 10.1186/1752-0509-7-S4-S6

**Published:** 2013-10-23

**Authors:** Yifeng Li, Alioune Ngom

**Affiliations:** 1 School of Computer Science, University of Windsor, Windsor, Ontario, Canada

## Abstract

**Background:**

High-throughput genomic and proteomic data have important applications in medicine including prevention, diagnosis, treatment, and prognosis of diseases, and molecular biology, for example pathway identification. Many of such applications can be formulated to classification and dimension reduction problems in machine learning. There are computationally challenging issues with regards to accurately classifying such data, and which due to dimensionality, noise and redundancy, to name a few. The principle of sparse representation has been applied to analyzing high-dimensional biological data within the frameworks of clustering, classification, and dimension reduction approaches. However, the existing sparse representation methods are inefficient. The kernel extensions are not well addressed either. Moreover, the sparse representation techniques have not been comprehensively studied yet in bioinformatics.

**Results:**

In this paper, a Bayesian treatment is presented on sparse representations. Various sparse coding and dictionary learning models are discussed. We propose fast parallel active-set optimization algorithm for each model. Kernel versions are devised based on their dimension-free property. These models are applied for classifying high-dimensional biological data.

**Conclusions:**

In our experiment, we compared our models with other methods on both accuracy and computing time. It is shown that our models can achieve satisfactory accuracy, and their performance are very efficient.

## Introduction

The studies in biology and medicine have been revolutionarily changed since the invents of many high-throughput sensory techniques. Using these techniques, the molecular phenomenons can be probed with a high resolution. In the virtue of such techniques, we are able to conduct systematic genome-wide analysis. In the last decade, many important results have been achieved by analyzing the high-throughput data, such as microarray gene expression profiles, gene copy numbers profiles, proteomic mass spectrometry data, next-generation sequences, and so on.

On one hand, biologists are enjoining the richness of their data; one another hand, bioinformaticians are being challenged by the issues of the high-dimensional data. Many of the analysis can be formulated into machine learning tasks. First of all, we have to face to the cures of high dimensionality which means that many machine learning models can be overfitted and therefore have poor capability of generalization. Second, if the learning of a model is sensitive to the dimensionality, the learning procedure could be extremely slow. Third, many of the data are very noise, therefore the robustness of a model is necessary. Forth, the high-throughput data exhibit a large variability and redundancy, which make the mining of useful knowledge difficult. Moreover, the observed data usually do not tell us the key points of the story. We need to discover and interpret the latent factors which drive the observed data.

Many of such analysis are classification problem from the machine learning viewpoint. Therefore in this paper, we focus our study on the classification techniques for high-dimensional biological data. The machine learning techniques addressing the challenges above can be categorized into two classes. The first one aims to directly classify the high-dimensional data while keeping the good capability of generalization and efficiency in optimization. The most popular method in this class is the *regularized basis-expended linear model*. One example is the state-of-the-art *support vector machine *(SVM) [1]. SVM can be kernelized and its result is theoretically sound. Combining different regularization terms and various loss functions, we can have many variants of such linear models [2]. In addition to classification, some of the models can be applied to regression and feature (biomarker) identification. However, most of the learned linear models are not interpretable, while interpretability is usually the requirement of biological data analysis. Moreover, linear models can not be extended *naturally *to multi-class data, while in bioinformatics a class may be composed of many subtypes.

Another technique of tackling with the challenges is dimension reduction which includes feature extraction and feature selection. *Principal component analysis *(PCA) [3] is the oldest feature extraction method and is widely used in processing high-dimensional biological data. The basis vectors produced by PCA are orthogonal, however many patterns in bioinformatics are not orthogonal at all. The classic *factor analysis *(FA) [4] also has orthogonal constraints on the basis vectors, however its Bayesian treatment does not necessarily produce orthogonal basis vectors. Bayesian factor analysis will be introduced in the next section.

*Sparse representation *(SR) [5] is a parsimonious principle that a sample can be approximated by a sparse linear combination of basis vectors. Non-orthogonal basis vectors can be learned by SR, and the basis vectors may be allowed to be redundant. SR highlights the parsimony in representation learning [6]. This simple principle has many strengthes that encourage us to explore its usefulness in bioinformatics. First, it is very robust to redundancy, because it only select few among all of the basis vectors. Second, it is very robust to noise [7]. Furthermore, its basis vectors are non-orthogonal, and sometimes are interpretable due to its sparseness [8]. There are two techniques in SR. First, given a basis matrix, learning the sparse coefficient of a new sample is called *sparse coding*. Second, given training data, learning the basis vector is called *dictionary learning*. As dictionary learning is, in essence, a sparse matrix factorization technique, *non-negative matrix factorization *(NMF) [9] can be viewed a specific case of SR. For understanding sparse representation better, we will give the formal mathematical formulation from a Bayesian perspective in the next section.

This paper is the significant extension of our preliminary work presented in [10] where sparse representation is treated from regularization and optimization perspectives. In this paper, we formulate sparse representation from a Bayesian viewpoint. We show that using different prior distributions, we can obtain various sparse coding and dictionary learning models. Although there exists some works, for example [11], which apply sparse coding in the classification of biological data, to the best of our knowledge, this is the first time that sparse representation is intensively and systematically studied in the area of bioinformatics. This study has the following contributions:

1. We give a Bayesian treatment on the sparse representation, which is very helpful to understand and design sparse representation models.

2. Kernel sparse coding techniques are proposed for direct classification of high-dimensional biological data.

3. Fast parallel active-set algorithms are devised for sparse coding.

4. An efficient generic framework of kernel dictionary learning for feature extraction is proposed.

5. We reveal that the optimization and decision making in sparse representation is *dimension-free*.

We organize the rest of this paper as follow. We first introduce factor analysis and sparse representation from a Bayesian aspect. Classification method based on sparse coding is then introduced and the active-set methods are proposed for the optimization. Their kernel extensions are given as well. Then various dictionary learning models are given. After that, a generic optimization framework is devised to optimize these models. In the same section, dictionary-learning-based classification and its kernel extension are proposed as well. Then we describe our computational experiments on two high-dimensional data sets. Finally, conclusions and future works are drawn.

## Related work from a Bayesian viewpoint

Both (sparse) factor analysis and sparse representation models can be used as dimension reduction techniques. Due to their intuitive similarity, it is necessary to give their definitions for comparison. In this section, we briefly survey the sparse factor analysis and sparse representation in a Bayesian viewpoint. The introduction of sparse representation is helpful to understand the content of the subsequent sections. Hereafter, we use the following notations unless otherwise stated. Suppose the training data is $D\in {\mathbb{R}}^{m\times n}$ (*m *is the number of features and *n *is the number of training instances (samples or observations)), the class information is in the *n*-dimensional vector ***c***. Suppose *p *new instances are in $B\in {\mathbb{R}}^{m\;\times p}$.

### Sparse Bayesian (latent) factor analysis

The advantages of *Bayesian (latent) factor analysis model *[12] over likelihood (latent) factor analysis model are that

1. The knowledge regarding the model parameters from experts and previous investigations can be incorporated through the prior.

2. The values of parameters are refined using the current training observations.

The Bayesian factor analysis model [12] can be formulated as

(1) (b|μ,A,x,k)=μ+Ax+ε,

where $b\in {\mathbb{R}}^{m\;\times \;1}$ is an observed multivariate variable, $\mu \in {\mathbb{R}}^{m\;\times \;1}$ is the population mean, $A\in {\mathbb{R}}^{m\;\;\times \;k}$ is *latent factor loading matrix*, and $x\in {\mathbb{R}}^{k\;\times \;1}$ is *latent factor score *(*k *≪ *m*), and $\varepsilon \in {\mathbb{R}}^{m\;\times \;1}$ is an idiosyncratic *error *term. This model is restricted by the following constraints or assumptions:

1. The error term is normally distributed with mean **0 **and covariance **Φ**: ***ε ***~ *N *(**0**, **Φ**). **Φ **is diagonal on average.

2. The factor score vector is also normally distributed with mean **0 **and identity covariance ***R ***= ***I***: ***x ***~ *N *(**0**, ***R***); and the factor loading vector is normally distributed: ***a***_*i *_~ *N *(**0**, **Δ**) where **Δ **is diagonal. Alternatively, the factor loading vectors can be normally distributed with mean **0 **and identity covariance **Δ **= ***I***; and the factor score vector is normally distributed with mean **0 **and diagonal covariance ***R***. The benefit of identity covariance either on ***x ***or ***A ***is that arbitrary scale interchange between ***A ***and ***x ***due to scale invariance can be avoided.

3. ***x ***is independent of ***ε***.

For *n *training instances ***D***, we have the likelihood:

(2) $$p\left(D|\mu ,A,Y,\Phi ,k\right)=\frac{1}{{\left(2\pi \right)}^{\frac{m\;n}{2}}|\Phi {|}^{\frac{n}{2}}}e^{-\frac{1}{2}\sum_{i=1}^{n}{\left(d_{i}-\mu -Ay_{i}\right)}^{\text{T}}\Phi^{-1}\left(d_{i}-\mu -Ay_{i}\right)}$$

(3) $$=\frac{1}{{\left(2\pi \right)}^{\frac{mn}{2}}|\Phi {|}^{\frac{n}{2}}}e^{-\frac{1}{2}\text{tr[}{\left(D-{\mu 1}^{\text{T}}-AY\right)}^{\text{T}}\Phi^{-1}(D-\mu 1^{\text{T}}-AY)]},$$

where tr(***M***) is the trace of square matrix ***M***.

The variants of Bayesian factor analysis models differ in the decomposition of the joint priors. The simplest one may be *p*(***µ***, ***A**, **Y***) = *p*(***µ***)*p*(***A***)*p*(***Y***). Suppose *k *is fixed a priori. The posterior therefore becomes

(4) p(μ,A,Y|D, k)∝p(D|μ,A,Y,Φ, k)p(μ)p(A)p(Y).

The model parameters are usually estimated via *Markov chain Monte Carlo *(MCMC) techniques.

*Sparse Bayesian factor analysis *model imposes a sparsity-inducing distribution over the factor loading matrix instead of Gaussian distribution. In [13], the following mixture of prior is proposed:

(5) $$p\left(a_{ij}\right)=\left(1-\pi_{ij}\right)\delta_{0}\left(a_{ij}\right)+\pi_{ij}N\left(a_{ij}|0,1\right),$$

where *π_ij _*is the probability of a nonzero *a_ij _*and *δ*_0_(·) is the Dirac delta function at 0. Meanwhile, ***A***is constrained using the lower triangular method. *Bayesian factor regression model *(BFRM) is the combination of Bayesian factor analysis and Bayesian regression [13]. It has been applied in oncogenic pathway studies [4] as a variable selection method.

### Sparse representation

*Sparse representation *(SR) is a principle that a signal can be approximated by a sparse linear combination of dictionary atoms [14]. The SR model can be formulated as

(6) $$\begin{array}{ccc} \left(b|A,x,k\right) & =x_{1}a_{1}+\cdots +x_{k}a_{k}+\varepsilon & \\ & =Ax+\varepsilon , \end{array}$$

where $A=\left[a_{1},\cdots \;,a_{k}\right]$ is called *dictionary*, ***a***_*i *_is a dictionary atom, ***x ***is a sparse *coefficient vector*, and ***ε ***is an error term. ***A***, ***x***, and *k *are the model parameters. SR model has the following constraints:

1. The error term is Gaussian distributed with mean zero and isotropic covariance, that is ***ε ***~ *N *(**0**, **Φ**) where **Φ **= *ϕ**I ***where ϕ is a positive scalar.

2. The dictionary atoms is usually Gaussian distributed, that is ***a***_i _~ *N *(**0**, **Δ**) where **Δ **= ***I***. The coefficient vector should follows a sparsity-inducing distribution.

3. ***x ***is independent of ***ε***.

Through comparing the concepts of Bayesian factor analysis and Bayesian sparse representation, we can find that the main difference between them is that the former applies a sparsity-inducing distribution over the factor loading matrix, while the later uses a sparsity-inducing distribution on the factor score vector.

Sparse representation involves sparse coding and dictionary learning. Given a new signal ***b ***and a dictionary ***A***, learning the sparse coefficient ***x ***is termed *sparse coding*. It can be statistically formulated as

(7) (b|A)=Ax+ε.

Suppose the coefficient vector has Laplacian prior with zero mean and isotropic variance, that is $p\left(x|\Gamma \right)=L\left(0,\Gamma \right)=\frac{1}{{\left(2\gamma \right)}^{k}}e^{-\frac{{||x||}_{1}}{\gamma }}$. The likelihood is Gaussian distributed as $p\left(b|A,x,\Phi \right)=N\left(Ax,\Phi \right)=\frac{1}{{\left(2\pi \right)}^{\frac{m}{2}}\phi^{\frac{m}{2}}}e^{-\frac{1}{2\phi }{||b,-,A,x||}_{2}^{2}}$. The posterior is thus

(8) $$\begin{array}{ccc} p\left(x|A,b,\Phi ,\Gamma \right) & =\frac{p\left(b|A,x,\Phi ,\Gamma \right)p\left(x|A,\Phi ,\Gamma \right)}{p\left(b\right)} & \\ & \propto p\left(b|A,x,\Phi \right)p\left(x|\Gamma \right). \end{array}$$

Taking the logarithm, the above is thus

(9) $$\begin{array}{ll} L\left(x\right) & =\text{log}p\left(b|A,x\right)+\text{log}p\left(x\right)\\ & =-\frac{1}{2\phi }{||b,-,A,x||}_{2}^{2}-\frac{{||x||}_{1}}{\gamma }+c, \end{array}$$

where *c *is a constant term. We can see that maximizing the posterior is equivalent to minimizing the following task:

(10) $$\underset{x}{\text{min}}f\left(x\right)=\frac{1}{2}{||b,-,A,x||}_{2}^{2}+\lambda {||x||}_{1},$$

where $\lambda =\frac{\phi }{\gamma }$. Hereafter we call Equation (10) *l*_1_-*least squares *(*l*_1 _LS) sparse coding model. It is known as the *l*_1_-regularized regression model in regularization theory. It coincides with the well-known *LASSO *model [15], which in fact is a *maximum a posteriori *(MAP) estimation.

Given training data ***D***, learning (or estimating) the dictionary ***A***, the coefficient vectors ***Y***, and the number of dictionary atoms *k *is called *dictionary learning*. Suppose *k *is given a priori, and consider the Laplacian prior over ***Y***and the Gaussian prior over ***A***, and suppose $p\left(A,Y\right)=p\left(A\right)p\left(Y\right)=\overset{k}{\underset{i=1}{\prod }}\left(p\left(a_{i}\right)\right)\overset{n}{\underset{i=1}{\prod }}\left(p\left(y_{i}\right)\right)$. We thus have the prior:

(11) $$p\left(A,Y|\Delta ,\Gamma \right)=\frac{1}{{\left(2\pi \right)}^{\frac{k}{2}}}e^{\sum_{i\;=\;1}^{k}-\frac{1}{2}\;{||a_{i}||}_{2}^{2}}\frac{1}{{\left(2\gamma \right)}^{kn}}e^{\sum_{i\;=\;1}^{n}-\frac{{||y_{i}||}_{1}}{\gamma }}$$

The likelihood is

(12) $$p\left(D|A,Y,\Phi \right)=\frac{1}{{\left(2\pi \right)}^{\frac{m\;n}{2}}\phi^{\;\frac{m\;n}{2}}}e^{-\;\frac{1}{2\phi }\text{tr}\left({||D,-,A,Y||}_{F}^{2}\right)}.$$

The posterior is

(13) $$p\left(A,Y|D,\Delta ,\Gamma ,\Phi \right)=\frac{p\left(D|A,Y,\Delta ,\Gamma ,\Phi \right)p\left(A,Y|\Delta ,\Gamma ,\Phi \right)}{p\left(D\right)}$$

(14) ∝p(D|A,Y,Φ)p(A|Δ)p(Y|Γ).

Ignoring the normalization term (that is the marginal likelihood), the log-posterior is thus

(15) $$L\left(A,Y\right)=-\overset{n}{\underset{i=1}{\sum }}\frac{1}{2\phi }{||d_{i},-,A,y_{i}||}_{2}^{2}-\overset{k}{\underset{i=1}{\sum }}\frac{1}{2}{||a_{i}||}_{2}^{2}-\overset{n}{\underset{i=1}{\sum }}\frac{{||y_{i}||}_{\;1}}{\gamma }+c.$$

Therefore the MAP estimation of dictionary learning task is

(16) $$\underset{A,Y}{\min}f\left(A,Y\right)=\overset{n}{\underset{i=1}{\sum }}\frac{1}{2}{||d_{i},-,A,y_{i}||}_{2}^{2}+\overset{k}{\underset{i=1}{\sum }}\frac{\alpha }{2}{||a_{i}||}_{2}^{2}+\overset{n}{\underset{i=1}{\sum }}\lambda {||y_{i}||}_{\;1},$$

where *α *= *f *and $\lambda =\frac{\phi }{\gamma }$. Equation (16) is known as a dictionary learning model based on *l*_1_-regularized least squares.

In the literature, the kernel extension of sparse representation is realized in two ways. The first way is to use empirical kernel in sparse coding as in [16], where dictionary learning is not considered. The second way is the one proposed in [17], where dictionary learning is involved. However, the dictionary atoms are represented and updated explicitly. This could be intractable, as the number of dimensions of dictionary atoms in the feature space is very high even infinite. In the later sections, we give our kernel extensions of sparse coding and dictionary learning, respectively, where any kernel functions can be used and the dictionary is updated efficiently.

## Sparse coding methods

Since, the *l*_1_LS sparse coding (Equation (10)) is a two-sided symmetric model, thus a coefficient can be zero, positive, or negative [18]. In Bioinformatics, *l*_1_LS sparse coding has been applied for the classification of microarray gene expression data in [11]. The main idea is in the following. First, training instances are collected in a dictionary. Then, a new instance is regressed by *l*_1_LS sparse coding. Thus its corresponding sparse coefficient vector is obtained. Next, the regression residual of this instance to each class is computed, and finally this instance is assigned to the class with the minimum residual.

We generalize this methodology in the way that the sparse code can be obtained by many other regularization and constraints. For example, we can pool all training instances in a dictionary (hence *k *= *n *and ***A ***= ***D***), and then learn the non-negative coefficient vectors of a new instance, which is formulated as an one-sided model:

(17) $$\underset{x}{\text{min}}\frac{1}{2}{||b,-,A,x||}_{2}^{2}\;\;\text{s}.\text{t}.\;x\geq 0.$$

We called this model the *non-negative least squares *(NNLS) sparse coding. NNLS has two advantages over *l*_1_LS. First, the non-negative coefficient vector is more easily interpretable than coefficient vector of mixed signs, under some circumstances. Second, NNLS is a non-parametric model. From a Bayesian viewpoint, Equation (17) is equivalent to the MAP estimation with the same Gaussian error as in Equation (6), but the following discrete prior:

(18) $$Pr\left(x\right)=\left\{\begin{array}{cc} 0.5^{k} & \text{if}x\geq 0,\\ 0 & \text{otherwise}. \end{array}\right)$$

This non-negative prior implies that, the elements in ***x ***are independent, and the probability of *x_i _*= 0 is 0.5 and the probability of *x_i _>*0 is 0.5 as well. (That is the probabilities of *x_i _*being either 0 or positive are equal, and the probability of being negative is zero.) Inspired by many sparse NMFs, *l*_1_-regularization can be additionally used to produce more sparse coefficients than NNLS above. The combination of *l*_1_-regularization and non-negativity results in the *l*_1_NNLS sparse coding model as formulated below:

(19) $$\underset{x}{\text{min}}\frac{1}{2}{||b,-,A,x||}_{2}^{2}+\lambda^{T}x\;\text{s}.\text{t}.\;x\geq 0.$$

We call Equation (19) the *l*_1_*NNLS *model. It is more flexible than NNLS, because it can produce more sparse coefficients as controlled by *λ*. This model in fact uses the following prior:

(20) $$p\left(x\right)=\left\{\begin{array}{cc} \frac{1}{\gamma^{k}}e^{-\frac{∥x{∥}_{\;1}}{\gamma }} & \text{if}\;x\geq 0,\\ 0 & \text{otherwise}. \end{array}\right)$$

Now, we give the generalized sparse-coding-based classification approach in details. The method is depicted in Algorithm 1. We shall give the optimization algorithms, later, required in the first step. The NN rule mentioned in Algorithm 1 is inspired by the usual way of using NMF as a clustering method. Suppose there are *C *classes with labels 1, ⋯, *C*. For a given new instance ***b***, its class is *l *= arg max_*i* = 1,⋯, *k *_x_*i*_. It selects the maximum coefficient in the coefficient vector, and then assigns the class label of the corresponding training instance to this new instance. Essentially, this rule is equivalent to applying *nearest neighbor *(NN) classifier in the column space of the training instances. In this space, the representations of the training instances are identity matrix. The NN rule can be further generalized to the weighted *K*-*NN *rule. Suppose a *K*-length vector $\bar{x}$ accommodates the *K*-largest coefficients from *x*, and $\bar{c}$ has the corresponding *K *class labels. The class label of ***b ***can be designated as *l *= arg max_*i *= 1, ⋯, *C *_*s*_i _where $s_{i}=\text{sum}(\delta_{i}(\bar{x}))$. $\delta_{i}\left(\bar{x}\right)$ is a *K*-length vector and is defined as

(21) $${\left(\delta_{i}\left(\bar{x}\right)\right)}_{j}=\left\{\begin{array}{cc} {\bar{x}}_{j} & \text{if}\;{\bar{c}}_{j}=i,\\ 0 & \text{otherwise}. \end{array}\right)$$

The maximum value of *K *can be *k*, the number of dictionary atoms. In this case, *K *is in fact the number of all non-zeros in ***x***. Alternatively, the *nearest subspace *(NS) rule, proposed in [19], can be used to interpret the sparse coding. NS rule takes the advantage of the discrimination of property in the sparse coefficients. It assigns the class with the minimum regression residual to ***b***. Mathematically, it is expressed as *j *= min_1*≤i≤C*_*r*_*i*_(***b***) where *r_i _*(***b***) is the regression residual corresponding to the *i*-th class and is computed as $r_{i}\left(b\right)\;=\;{||b,-,A,\delta_{i},\left(x\right)||}_{2}^{2}$, where ***δ***_*i*_(***x***) is defined analogically as in Equation (21).

**Algorithm 1 ***Sparse-coding-based classification*

**Input**: ***A***_*m×n*_: *n *training instances, ***c***: class labels, ***B***_*m×p*_: *p *new instances

**Output**: ***p***: predicted class labels of the *p *new instances

1. Normalize each instance to have unit *l*_2_-norm.

2. Learn the sparse coefficient matrix ***X***, of the new instances by solving Equation (10), (17), or (19).

3. Use a sparse interpreter to predict the class labels of new instances, e.g. the NN, *K*-NN, or NS rule.

### Optimization

#### Active-set algorithm for l_1 _LS

The problem in Equation (10) is equivalent to the following non-smooth unconstrained *quadratic programming *(QP):

(22) $$\underset{x}{\text{min}}\frac{1}{2}x^{\text{T}}Hx+g^{\text{T}}x+\lambda {∥x∥}_{1},$$

where ***H***_*k×k *_= ***A***^T^***A***, and ***g ***= *-**A***^T^***b***. We thus know that the *l*_1_LS problem is a *l*_1_QP problem. This can be converted to the following smooth constrained QP problem:

(23) $$\underset{x,u}{\text{min}}\frac{1}{2}x^{\text{T}}Hx+g^{\text{T}}x+\lambda^{\text{T}}u\;\text{s}.\text{t}.\;-u\leq x\leq u,$$

where ***u ***is an auxiliary vector variable to squeeze ***x ***towards zero. It can be further written into the standard form:

(24) $$\underset{x,u}{\text{min}}\frac{1}{2}\left[x^{\text{T}},u^{\text{T}}\right]\left[\begin{array}{c} H\\ 0_{k\times k} \end{array}\begin{array}{c} 0_{k\times k}\\ 0_{k\times k} \end{array}\right]\;\left[\begin{array}{c} x\\ u \end{array}\right]+g^{\text{T}}x+\lambda^{\text{T}}u$$

$$s.t.\left[\begin{array}{c} I_{k\times k}\\ -I_{k\times k} \end{array}\begin{array}{c} -I_{k\times k}\\ -I_{k\times k} \end{array}\right]\leq 0,$$

where ***I ***is an identity matrix. Obviously, the Hessian in this problem is positive semi-definite as we always suppose ***H ***is positive semi-definite in this paper.

A general active-set algorithm for constrained QP is provided in [20], where the main idea is that a working set is updated iteratively until it meets the true active set. In each iteration, a new solution ***x***_t _to the QP constrained only by the current working set is obtained. If the update step ***p***_t _= ***x***_t _- **x**_*t-*1 _is zero, then Lagrangian multipliers of the current active inequalities are computed. If all these multipliers corresponding to the working set are non-negative, then the algorithm terminates with an optimal solution. Otherwise, an active inequality is dropped from the current working set. If the update step ***p***_t _is nonzero, then an update length *α *is computed using the inequality of the current passive set. The new solution is updated as ***x***_*t *_= ***x***_*t-*1 _+ *α**p**_t_*. If *α <*1, then a blocking inequality is added to the working set.

To solve our specific problem efficiently in Equation (24), we have to modify the general method, because i) our constraint is sparse, for the *i*-th constraint, we have *x_i _- u_i _≤ *0 (if *i ≤ k*) or *-x_i _- u_i _≤ *0 (if *i > k*); and ii) when *u*_*i *_is not constrained in the current working set, the QP constrained by the working set is unbounded, therefore it is not necessary to solve this problem to obtain ***p***_*t*_. In the later situation, ***p***_t _is unbounded. This could cause some issues in numerical computation. Solving the unbounded problem is time-consuming if the algorithm is unaware of the unbounded issue. If ***p***_*t *_contains positive or negative *∞*, then the algorithm may crash.

We propose the revised active-set algorithm in Algorithm 2 for *l*_1_LS sparse coding. To address the potential issues above, we have the following four modifications. First, we require that the working set is complete. That is all the variables in ***u ***must be constrained when computing the current update step. (And therefore all variables in ***x ***are also constrained due to the specific structure of the constraints in our problem.) For example, if *k *= 3, a working set {1, 2, 6} is complete as all variables, *x*_1_, *x*_2_, *x*_3_, *u*_1_, *u*_2_, *u*_3_, are constrained, while {1, 2, 4} is not complete, as *u*_3 _(and *x*_3_) is not constrained. Second, the update step of the variables that are constrained once in the working set are computed by solving the equality constrained QP. The variables constrained twice are directly set to zeros. In the example above, suppose the current working set is {1, 2, 4, 6}, then *x*_2_, *x*_3_, *u*_2_, *u*_3 _are computed by the constrained QP, while *x*_1 _and *u*_1 _are zeros. This is because the only value satisfying the constraint *-u*_1 _= *x*_1 _= *u*_1 _is *x*_1 _= *u*_1 _= 0. Third, in this example, we do not need to solve the equality constrained QP with four variables. In fact we only need two variables by setting *u*_2 _= *-x*_2 _and *u*_3 _= *x*_3_. Forth, once a constraint is dropped from the working set and it becomes incomplete, other inequalities must be immediately added to it until it is complete. In the initialization of Algorithm 2, we can alternatively initialize ***x ***by 0's. This is much efficient than ***x ***= (***H***)^-1^(*-**g***) for large-scale sparse coding and very sparse problems.

#### Active-set algorithm for NNLS and l*_1 _*NNLS

Both the NNLS problem in Equation (17) and the *l*_1_NNLS problem in Equation (19) can be easily reformulated to the following *non-negative QP *(NNQP) problem:

(26) $$\text{min}\frac{1}{2}x^{\text{T}}H\;x+g^{\text{T}}x\;\text{s}.\text{t}.\;x\geq 0,$$

**Algorithm 2 ***Active-set l*_1_*QP algorithm*

**Input**: Hessian ***H***_*k×k*_, vector ***g***_*k×*1_, scalar *λ*

**Output**: vector ***x ***which is a solution to min $\frac{1}{2}x^{\text{T}}Hx+g^{\text{T}}x+\lambda^{\text{T}}u,\text{s}.\text{t}.-u\leq x\leq u$

 % *initialize the algorithm by a feasible solution and complete working set*

 *x *= (***H***)^-1^(*-**g***); ***u ***= *|**x**|*;

 $R=\left\{i,\;j|\forall i:\text{if}\;x_{i}>0\text{ let}\;j=k+i\;\text{otherwise }j=i\right\}$; % initialize working set

 P={1:2k}-R; % initialize inactive(passive) set

 **while **true **do**

  % *compute update step*

  let $R_{\text{once}}$ be the indices of variables constrained once by  R;

  ***p***_2*k×*1 _= 0;

  $p_{x,R_{\text{once}}}=\text{arg}\;{\text{min}}_{q}q^{\text{T}}H_{R_{\text{once}}}q+{\left[H_{R_{\text{once}}}{x_{R}}_{{}_{\text{once}}}+g_{R_{\text{once}}}+\lambda e\right]}^{\text{T}}q$ where *e_i _*= 1 if $u_{R_{\text{once}},i}=x_{R_{\text{once}},i}$, or -1 if $u_{R_{\text{once}},i}=x_{R_{\text{once}},i}$;

  **if *p ***= 0 **then**

   obtain Lagrange multiplier ***µ ***by solving

(25) $$A_{R}^{T}\mu =-\left[\begin{array}{c} Hx+g\\ \lambda \end{array}\right]$$

   where ***A ***is the constraint matrix in Equation (24)

   **if **$\mu_{i}\geq 0\forall i\in R$**then**

    terminate successfully;

   **else**

    R=R-j;P=P+j where $\;j=\text{ arg mi}{\text{n}}_{l\in R}\mu_{l}$;

    add other passive constraints to  R until it is complete;

   **end if**

  **end if**

  **if *p ***≠ 0 **then**

   $\alpha =\text{min}\left(1,{\text{min}}_{i\in P,a_{i}^{\text{T}}p\geq 0}\frac{-a_{i}^{\text{T}}\left[x;u\right]}{a_{i}^{\text{T}}p}\right);$

   [***x***; ***u***] = [***x***; ***u***] + *α**p***;

   **if ***α <*1 **then**

    R=R+i; P=P-i. where *i *corresponds to *α*;

   **end if**

  **end if**

 **end while**

where ***H ***= ***A***^T^***A***, ***g ***= *-**A***^T^***b ***for NNLS, and ***g ***= *-**A***^T^***b ***+ ***λ ***for *l*_1_NNLS.

Now, we present the active-set algorithm for NNQP. This problem is easier to solve than *l*_1_QP as the scale of Hessian of NNQP is half that of *l*_1_QP and the constraint is much simpler. Our algorithm is obtained through generalizing the famous active-set algorithm for NNLS by [21]. The complete algorithm is given in Algorithm 3. The warm-start point is initialized by the solution to the unconstrained QP. As in Algorithm 2, ***x ***can be alternatively initialized by 0's. The algorithm keeps adding and dropping constraints in the working set until the true active set is found.

**Algorithm 3 ***Active-set NNQP algorithm*

**Input**: Hessian ***H***_*k×k*_, vector ***g***_*k×*1_

**Output**: vector ***x ***which is a solution to $\text{min}\frac{1}{2}x^{\text{T}}Hx+g^{\text{T}}x,\text{s}.\text{t}.\;x\geq 0$

 *x *= [(***H***)^-1^(*-**g***)]_+_; % ***x ***= [***y***]_+_*is defined as x_i _*= *y_i _if y_i _>*0*, otherwise x_i _*= 0

 $R=\left\{i|x_{i}=\;0\right\}$; % *initialize active set*

 $P=\left\{i|x_{i}>0\right\}$; % *initialize inactive(passive) set*

 ***µ ***= ***Hx ***+ ***g***; % *the lagrange multiplier*

 **while *R ***¹ **Æ **and min_*i*Î*R *_(*µ_i_*) *< -e ***do**

  % *e is a small positive numerical tolerance*

  *j *= arg min_*i*Î*R *_(*µ_i_*); % *get the minimal negative multiplier*

  P=P+{j};R=R-{j};

  $t_{P}={\left(H_{P}\right)}^{-1}\left(-g_{P}\right);$

  $t_{R}=0;$

  **while **min $t_{P}\leq 0$**do**

   $\alpha =\underset{i\in P,t_{i}\leq 0}{\text{min}}\frac{x_{i}}{x_{i}-t_{i}};$

   $K=\text{arg}\;{\text{min}}_{i\in P,t_{i}\leq 0}\frac{x_{i}}{x_{i}-t_{i}}$; % *there is one or several indices correspond to α*

   *x *= *x *+ *α*(*t *- *x*);

   P=P-K;R=R+K;

   $t_{P}={\left(H_{P}\right)}^{-1}\left(-g_{P}\right);$

   $t_{R}=0;$

  **end while**

  *x *= *t*;

  *µ *= *Hx *+ *g*;

 **end while**

#### Parallel active-set algorithms

The formulations of *l*_1_QP and NNQP sparse coding for *p *new instances are, respectively,

(27) $$\underset{X,U}{\text{min}}\overset{p}{\underset{i=1}{\sum }}\frac{1}{2}x_{i}^{\text{T}}Hx_{i}+g_{i}^{\text{T}}x_{i}+\lambda^{\text{T}}u_{i},$$

s.t.-U≤X≤U,

and

(28) $$\underset{X}{\text{min}}\overset{p}{\underset{i=1}{\sum }}\frac{1}{2}x_{i}^{\text{T}}Hx_{i}+g_{i}^{\text{T}}x_{i}\;\text{s}.\text{t}.\;X\geq 0.$$

If we want to classify multiple new instances, the initial idea in [19] and [11] is to optimize the sparse coding one at a time. The interior-point algorithm, proposed in [22], is a fast large-scale sparse coding algorithm, and the proximal algorithm in [23] is a fast first-order method whose advantages have been recently highlighted for non-smooth problems. If we adapt both algorithms to solve our multiple *l*_1_QP in Equation (27) and NNQP in Equation (28), it will be difficult to solve the single problems in parallel and share computations. Therefore, the time-complexity of the multiple problems will be the summation of that of the individual problems. However, the multiple problems can be much more efficiently solved by active-set algorithms. We adapt both Algorithms 2 and 3 to solve multiple *l*_1_QP and NNQP in a parallel fashion. The individual active-set algorithms can be solved in parallel by sharing the computation of matrix inverses (systems of linear equations in essence). At each iteration, single problems having the same active set have the same systems of linear equations to solve. These systems of linear equations can be solved once only. For a large value *p*, that is large-scale multiple problems, active-set algorithms have dramatic computational advantage over interior-point [22] and proximal [23] methods unless these methods have a scheme of sharing computations. Additionally, active-set methods are more precise than interior-point methods. Interior-point methods do not allow $u_{i}^{2}=x_{i}^{2}$ and $u_{i}^{2}$ must be always greater than $x_{i}^{2}$ due to feasibility. But $u_{i}^{2}=x_{i}^{2}$ is naturally possible when the *i*-th constraint is active. *u_i _*= *x_i _*= 0 is reasonable and possible. Active-set algorithms do allow this situation.

### Kernel extensions

As the optimizations of *l*_1_QP and NNQP only require inner products between the instances instead of the original data, our active-set algorithms can be naturally extended to solve the kernel sparse coding problem by replacing inner products with kernel matrices. The NS decision rule used in Algorithm 1 also requires only inner products. And the weighted *K*-NN rule only needs the sparse coefficient vector and class information. Therefore, the classification approach in Algorithm 1 can be extended to kernel version. For narrative convenience, we also denote the classification approaches using *l*_1_LS, NNLS, and *l*_1_NNLS sparse coding as *l*_1_LS, NNLS, and *l*_1_NNLS, respectively. Prefix "K" is used for kernel versions.

## Dictionary learning methods

We pursue our dictionary-learning-based approach for biological data, based on the following two motivations. First, since sparse-coding-only approach is a lazy learning, the optimization can be slow for large training set. Therefore, learning a concise dictionary is more efficient for future real-time applications. Second, dictionary learning may capture hidden key factors which correspond to biological pathways, and the classification performance may hence be improved. In the following, we first give the dictionary learning models using Gaussian prior and uniform prior, respectively. Next, we give the classification method based on dictionary learning. We then address the generic optimization framework of dictionary learning. Finally, we show that the kernel versions of our dictionary learning models and the classification approach can be easily obtained.

### Dictionary learning models

Now we give our dictionary learning models using Gaussian prior and uniform prior over the dictionary atoms, respectively. Both priors aims to get rid off the arbitrary scale interchange between dictionary and coefficient. Suppose ***D***_*m×n *_is the data of *n *training instances, and the dictionary ***A ***to be learned has *k *atoms. If the Gaussian prior in Equation (6) is used on the dictionary atom, our dictionary learning models of *l*_1_LS, NNLS, and *l*_1_NNLS are expressed as follow, respectively:

(29) $$l_{1}LS:\underset{A,Y}{\text{min}}\frac{1}{2}{∥D-AY∥}_{F}^{2}+\frac{\alpha }{2}\text{trace}\left(A^{\text{T}}A\right)+\lambda \overset{n}{\underset{i=1}{\sum }}{∥y_{i}∥}_{1},$$

(30) $$NNLS:\underset{A,Y}{\text{min}}\frac{1}{2}{∥D-AY∥}_{F}^{2}+\frac{\alpha }{2}\text{trace}\left(A^{\text{T}}A\right)$$

s.t.Y≥0,

and

(31) $$l_{1}NNLS:\underset{A,Y}{\text{min}}\frac{1}{2}{∥D-AY∥}_{F}^{2}+\frac{\alpha }{2}\text{trace}\left(A^{\text{T}}A\right)+\overset{n}{\underset{i=1}{\sum }}\lambda^{\text{T}}y_{i}$$

s.t.Y≥0.

The strength of the Gaussian prior based dictionary learning is that it is flexible to control the scales of dictionary atoms. However, it has two model parameters, which increase the model selection burden in practice. Alternatively, in order to eliminate the parameter *α*, we design an uniform prior over the dictionary which is expressed as

(32) $$Pr\left(a_{i}\right)=\left\{\begin{array}{cc} p & \text{if}{∥a_{i}∥}_{2}=1,\\ 0 & \text{otherwise}, \end{array}\right)$$

where *p *is a constant. That is the feasible region of the dictionary atoms is a hypersphere centered at origin with unit radius, and all the feasible atoms have equal probability. The corresponding dictionary learning models are given in the following equations, respectively:

(33) $$l_{1}LS:\underset{A,Y}{\text{min}}\frac{1}{2}{∥D-AY∥}_{F}^{2}+\lambda \overset{n}{\underset{i=1}{\sum }}{∥y_{i}∥}_{1}$$

$$\text{s}.\text{t}.\;a_{i}^{\text{T}}a_{i}=1,\;i=1,\cdots \;,k,$$

(34) $$NNLS:\underset{A\text{,}Y}{\text{min}}\frac{1}{2}{∥D-AY∥}_{F}^{2}$$

$$\text{s}.\text{t}.\;a_{i}^{\text{T}}a_{i}=1,\;\;i=1,\cdots \;,k;\;Y\geq 0,$$

and

(35) $$l_{1}NNLS:\underset{A,Y}{\text{min}}\frac{1}{2}{∥D-AY∥}_{F}^{2}+\overset{n}{\underset{i=1}{\sum }}\lambda^{\text{T}}y_{i}$$

$$\text{s}.\text{t}.\;a_{i}^{\text{T}}a_{i}=1,\;\;i=1,\cdots \;,k;\;\;\;Y\geq 0.$$

### A generic optimization framework for dictionary learning

We devise block-coordinate-descent-based algorithms for the optimization of the above six models. The main idea is that, in the next step, ***Y ***is fixed, and the inner product ***A***^T^***A***, rather than ***A ***itself, is updated; in the next step, ***Y ***is updated while fixing ***A***^T^***A ***(a sparse coding procedure). The above procedure is repeated until the termination conditions are satisfied.

Now, we show that ***A ***can be analytically obtained. For normal prior over dictionary atoms, the optimization of finding ***A ***in Equations (29), (30), and (31) is to solve

(36) $$\underset{A}{\text{min}}f\left(A\right)=\frac{1}{2}{∥D-AY∥}_{F}^{2}+\frac{\alpha }{2}\text{trace}\left(A^{\text{T}}A\right)$$

Taking the derivative with respect to ***A ***and setting it to zero, we have

(37) $$\frac{\partial f\left(A\right)}{\partial A}=AYY^{\text{T}}-DY^{\text{T}}+\alpha A=0.$$

We hence have

(38) $$A=DY^{‡},$$

where ***Y ***^‡ ^= ***Y ***^T^(***Y Y ***^T ^+ *α**I***)^-1^. The inner product ***A***^T^***A ***can thus be updated by

(39) $$R=A^{\text{T}}A=\;{\left(Y^{‡}\right)}^{\text{T}}D^{\text{T}}DY^{‡}.$$

We also can compute ***A***^T^***D ***by

(40) $$A^{\text{T}}D=\;{\left(Y^{‡}\right)}^{\text{T}}D^{\text{T}}D.$$

For the uniform prior as in Equation (32), updating unnormalized ***A ***while fixing ***Y ***in Equations (33), (34), and (35) is to solve the generalized least squares:

(41) $$\underset{A}{\text{min}}f\left(A\right)=\frac{1}{2}{∥D-AY∥}_{F}^{2}.$$

Taking derivative with respect to ***A ***and setting it to zero, we have

(42) $$A=DY^{†},$$

**Algorithm 4 **The generic dictionary learning framework

**Input: *K ***= ***D***^T^***D***, dictionary size *k, λ*

**Output: *R ***= ***A***^T^***A***, ***Y***

nitialize ***Y ***and ***R ***= ***A***^T^***A ***randomly;

*r_prev _*= *Inf *; % *previous residual*

**for ***i *= 1 : *maxIter ***do**

 update ***Y ***by solving the active-set based *l*_1_LS, NNLS, or *l*_1_NNLS sparse coding algorithms;

 **if **Gassian prior over ***A *then**

  update ***R ***= ***Y ***^‡T^***D***^T^***DY ***^‡^;

 **end if**

 **if **uniform prior over ***A *then**

  update ***R ***= ***Y ***^†T^***D***^T^***DY ***^†^;

  normalize ***R ***by $R=R./\sqrt{\text{diag}\left(R\right)\text{diag}{\left(R\right)}^{\text{T}}}$;

 **end if**

 **if ***i *== *maxIter *or *i *mod *l *= 0 **then**

  % *check every l iterations*

  *r_cur _*= *f *(***A***, ***Y ***); % *current residual of a dictionary learning model*

  **if ***r_prev _- r_cur _≤ e *or *r_cur _≤ e ***then**

   break;

  **end if**

  *r_prev _*= *r_cur_*;

 **end if**

end for

where ***Y ***^† ^= ***Y ***^T^(***Y Y ***^T^)^-1^. The inner products of ***R ***= ***A***^T^***A ***and ***A***^T^***D ***are computed similarly as for the Gaussian prior. The normalization of ***R ***is straightforward. We have $R=R./\sqrt{\text{diag}\left(R\right)\text{diag}{\left(R\right)}^{\text{T}}}$, where ./ and $\;\sqrt{•}$ are element-wise operators. Learning the inner product ***A***^T^***A ***instead of ***A ***has the benefits of dimension-free computation and kernelization.

Fixing ***A***, ***Y ***can be obtained via our active-set algorithms. Recall that the sparse coding only requires the inner products ***A***^T^***A ***and ***A***^T^***D***. As shown above, we find that updating ***Y ***only needs its previous value and the inner product between training instances.

Due to the above derivation, we have the framework of solving our dictionary learning models as illustrated in Algorithm 4.

### Classification approach based on dictionary learning

Now, we present the dictionary-learning-based classification approach in Algorithm 5. The dictionary learning in the training step should be consistent with the sparse coding in the prediction step. As discussed in the previous section, the sparse coding in the prediction step needs the inner products ***A***^T^***A***, ***B***^T^***B ***and ***A***^T^***B ***which actually is ***Y ***^‡T^***D***^T^***B ***or ***Y ***^‡T^***D***^T^***B***.

**Algorithm 5 ***Dictionary-learning-based classification*

**Input**: ***D***_*m×n*_: *n *training instances, ***c ***the class labels, ***B***_*m×p*_: *p *new instances, *k*: dictionary size

**Output**: ***p***: the predicted class labels of the *p *new instances

 **{training step:}**

 1: Normalize each training instance to have unit *l*_2 _norm.

 2: Learn dictionary inner product ***A***^T^***A ***and sparse coefficient matrix ***Y ***of training instances by Algorithm 4.

 3: Train a classifier *f *(*θ*) using ***Y ***(in the feature space spanned by columns of ***A***).

 **{prediction step:}**

 1: Normalize each new instance to have unit *l*_2 _norm.

 2: Obtain the sparse coefficient matrix ***X ***of the new instances by solving Equation (27), or (28).

 3: Predict the class labels of ***X ***using the classifier *f *(*θ*) learned in the training phase.

### Kernel extensions

For Gaussian dictionary prior, the *l*_1_LS based kernel dictionary learning and sparse coding are expressed in the following, respectively:

(43) $$\underset{A_{\phi },Y}{\text{min}}\frac{1}{2}{∥\phi \left(D\right)-A_{\phi }Y∥}_{F}^{2}+\frac{\alpha }{2}\text{trace}\left(A_{\phi }^{\text{T}}A_{\phi }\right)+\lambda {∥Y∥}_{1},$$

$$\underset{X}{\text{min}}\frac{1}{2}{∥\phi \left(B\right)-A\phi X∥}_{F}^{2}+\lambda {∥X∥}_{1},$$

where *f *(*·*) is a mapping function. Equations (30), (31), (33), (34), (35) and their sparse coding models can be kernelized analogically. As we have mentioned that the optimizations of the six dictionary learning models, only involves inner products of instances. Thus, we can easily obtain their kernel extensions by replacing the inner products with kernel matrices. Hereafter, if dictionary learning is employed in sparse representation, then prefix "DL" is used before "*l*_1_LS", "NNLS", and "*l*_1_NNLS". If kernel function other than the linear kernel is used in dictionary learning, then prefix "KDL" is added before them.

## Computational experiments

Two high-throughput biological data, including a microarray gene expression data set and a protein mass spectrometry data set, are used to test the performance of our methods. The microarray data set is a collection of gene expression profiles of breast cancer subtypes [24]. This data set includes 158 tumor samples from five subtypes measured on 13582 genes. The mass spectrometry data set is composed of 332 samples from normal class and prostate cancer class [25]. Each sample has 15154 features, that is the mass-to-charge ratios. Our experiments are separated into two parts. The performance of sparse coding for direct classification is first investigated with respect to accuracy and running time. Then our dimension reduction techniques using dictionary learning are tested.

### Sparse coding for direct classification

When dictionary learning was not involved, the dictionary was "lazily" composed by all the training instances available. In our experiment, the active-set optimization methods for *l*_1_LS, NNLS, and *l*_1_NNLS were tested. The weighted *K*-NN rule and NS rule, mentioned in Algorithm 1, were compared. We set *K *in the *K*-NN rule to the number of all training instances, which is an extreme case as opposite to the NN rule. Linear and *radial basis function *(RBF) kernels were employed. We compared our active-set algorithms with the interior-point [22] method and proximal [23] method for *l*_1_LS sparse coding (abbreviated by *l*_1_LS-IP and *l*_1_LS-PX). Benchmark classifiers, including *k*-NN and SVM using RBF kernel, were compared. We employed four-fold *cross-validation *to partition a data set into training sets and test sets. All the classifiers ran on the same training and test splits for fair comparison. We performed 20 runs of cross-validation and recorded the averages and standard deviations. Line or grid search was used to select the parameters of a classifiers.

**Figure 1:**
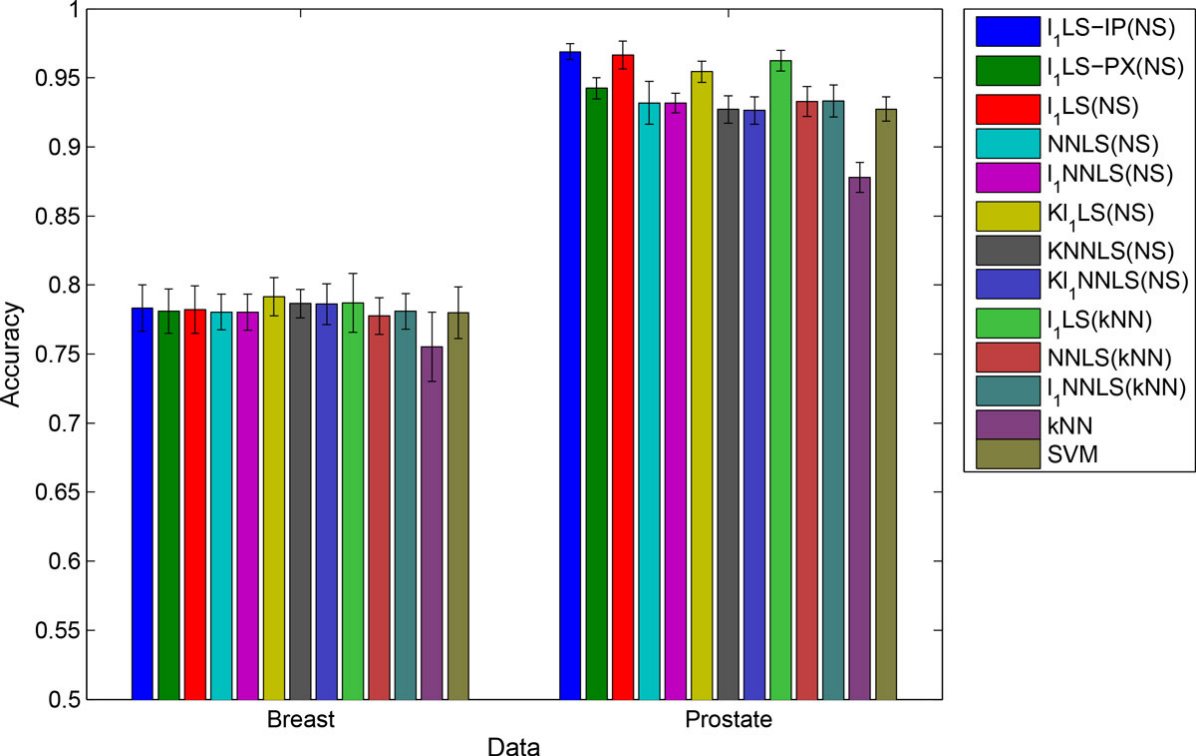
**Mean accuracies and standard deviations of sparse coding and benchmark methods**.

The average accuracies of all classifiers with the corresponding standard deviations on both data sets are compared in Figure 1, from which we have four observations. First, the weighted *K*-NN rule obtained comparable accuracies with the NS rule. The advantage of the *K*-NN rule over the NS rule is that the former predicts the class labels based on the sparse coefficient vector solely, while the later has to use the training data to compute regression residuals. Therefore the *K*-NN rule is more efficient and should be preferred. Second, on the Prostate data, the sparse coding method *l*_1_LS and K*l*_1_LS achieved the best accuracy. This convinces us that sparse coding based classifiers can be very effective for classifying high-throughput biological data. Third, the non-negative models including NNLS, *l*_1_NNLS and their kernel extensions achieved competitive accuracies with the state-of-the-art SVM on both data set. Fourth, the *l*_1_LS sparse coding using our active-set algorithm had the same accuracies as that using the interior-point algorithm and proximal algorithm on Breast data. But on Prostate data, the proximal method yielded a worse accuracy. This implies that our active-set method converges to the global minima as the interior-point method, while performance may be deteriorated by the approximate solution obtained by the proximal method in practice.

**Figure 2:**
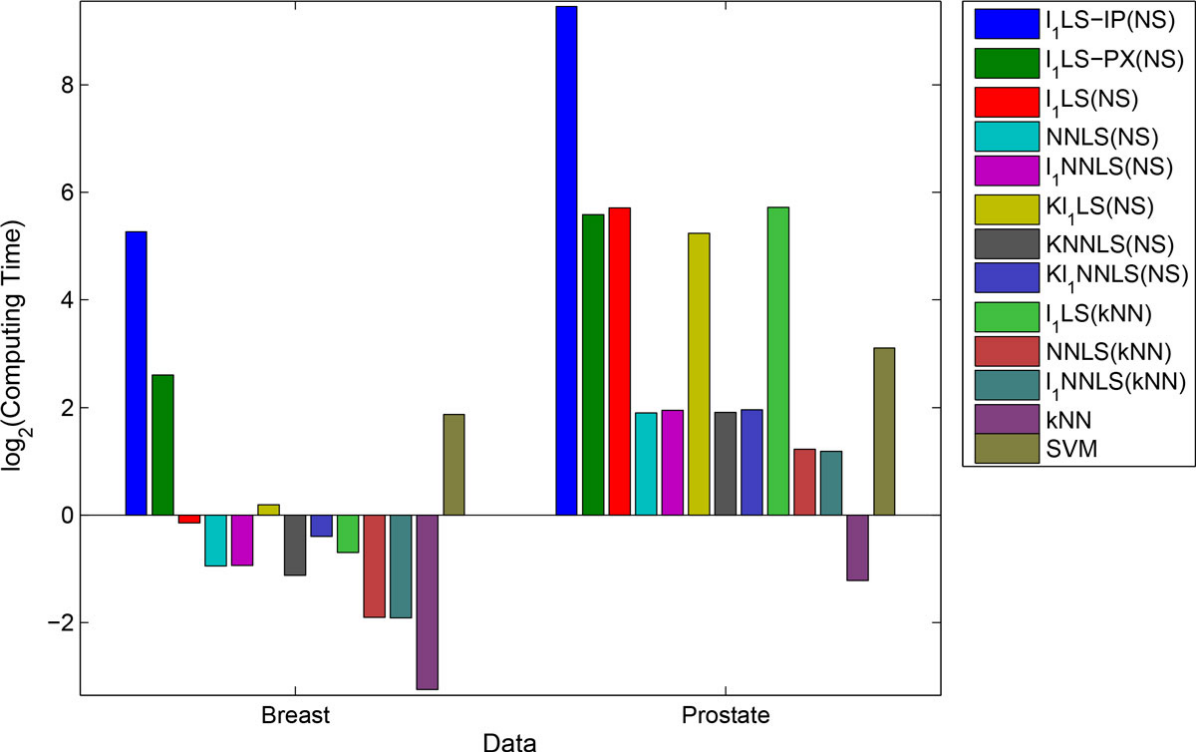
**Log2 computing time of sparse coding and benchmark methods**.

The mean running time (in second) of cross-validation are shown in Figure 2. For better comparison, logarithm of base two was taken on the results. First of all, we can clearly see that the interior-point method is very slow for the *l*_1_LS sparse coding. Second, our active-set method is more efficient than the proximal method on Breast data. This is because i) active-set methods are usually the fastest ones for quadratic and linear programmes of small and median size; and ii) expensive computations, like solving systems of linear equations, can be shared in the active-set method. Third, NNLS and *l*_1_NNLS have the same time-complexity. This is reasonable, because both can be formulated to NNQP problem. These non-negative models are much simpler and faster than the non-smooth *l*_1_LS model. Hence, if similar performance can be obtained by *l*_1_LS and the non-negative models in an application, we should give preference to NNLS and *l*_1_NNLS.

### Dictionary-learning for feature extraction

The performance of various dictionary learning models with linear and RBF kernels were investigated on both Breast and Prostate data sets. The Gaussian-prior based and uniform-prior based dictionary learning models were also compared. Again, our active-set dictionary learning was compared with the interior-point [22] method and proximal method [23]. The semi-NMF based on multiplicative update rules [26] is also included in the competition. As in the previous experiment, four-fold cross-validation was used. All methods ran on the same splits of training and test sets. We performed 20 runs of cross-validation for reliable comparison. After feature extraction by using dictionary learning on the training set, linear SVM classifier was learned on the reduced training set and used to predict the class labels of test instances.

**Figure 3:**
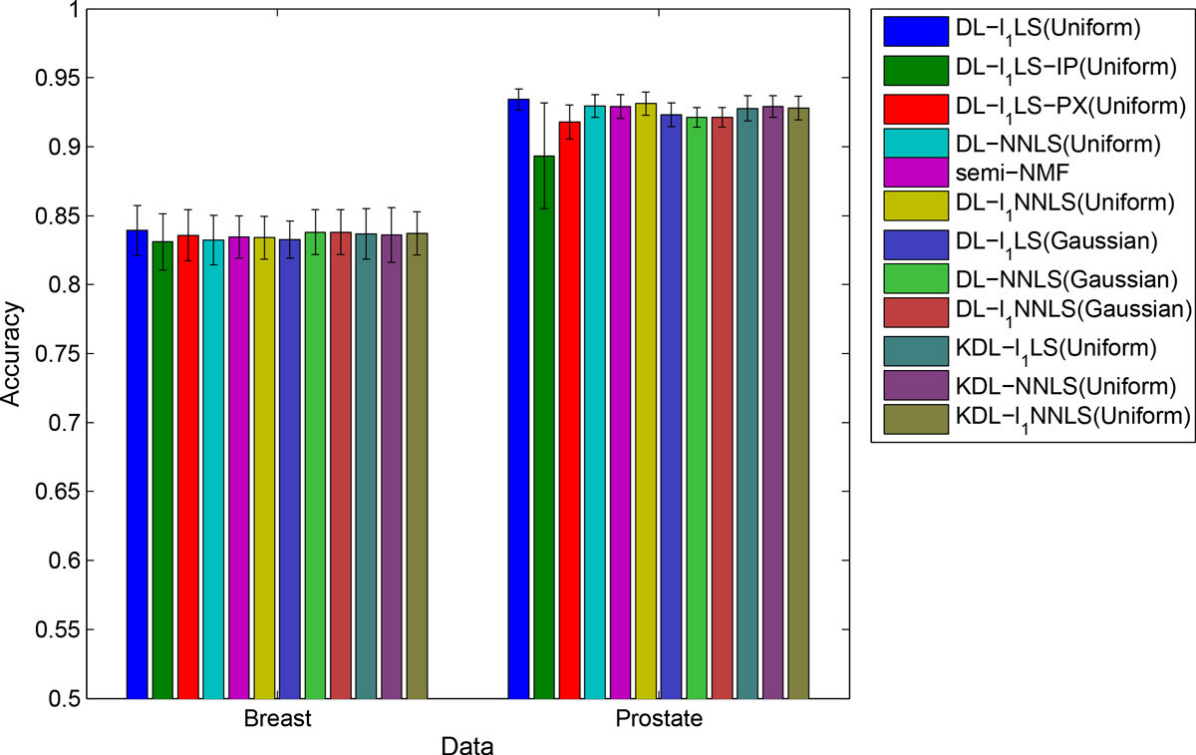
**Mean accuracies and standard deviations of dictionary learning methods**.

In Figure 3, we show the mean accuracy and standard deviation of 20 results for each method. First, we can see that the models with Gaussian prior on dictionary atoms obtained similar accuracies as the uniform prior. Second, with the comparison to sparse coding methods on Breast data as given Figure 1, we can see that dictionary learning increases the prediction accuracy. Third, from the comparison of Figures 3 and 1, we find that the dictionary learning based methods - DL-NNLS and DL-*l*_1_NNLS, obtained similar accuracies as the sparse coding methods - NNLS and *l*1NNLS. This convinces us that dictionary learning is a promising feature extraction technique for high-dimensional biological data. On Prostate data, we can also find that the accuracy obtained by DL-*l*_1_LS is slightly lower than *l*_1_LS. This is may be because the dictionary learning is unsupervised. Fourth, using the model parameters, DL-*l*_1_LS using active-set algorithm obtained higher accuracy than DL-*l*_1_LS-IP and DL-*l*_1_LS-PX on Prostate data. The accuracy of DL-*l*1LS is also slightly higher than that of DL-*l*_1_LS-IP on Breast data. Furthermore, the non-negative DL-NNLS yielded the same performance as the well-known semi-NMF, while further corroborates the satisfactory performance of our dictionary learning framework. Finally, the kernel dictionary learning models achieved similar performance as their linear counterparts. We believe that the accuracy could be further improved by a suitably selected kernel.

**Figure 4:**
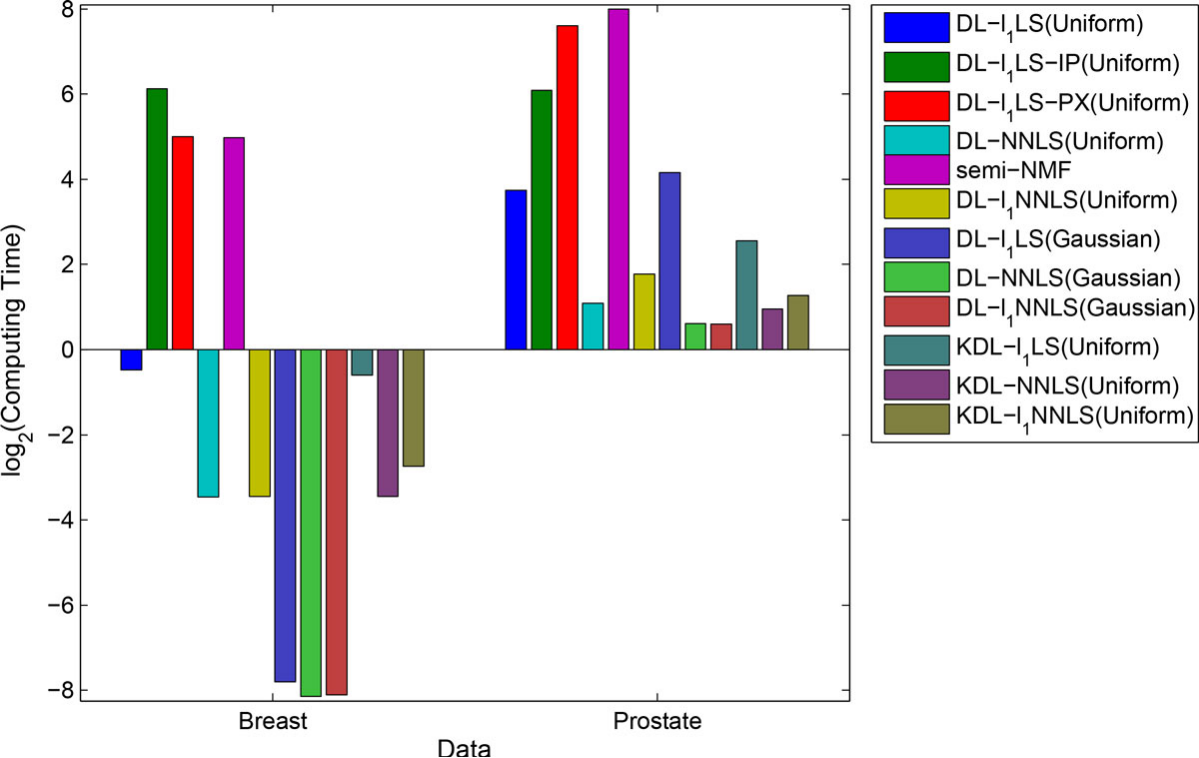
**Log2 computing time of dictionary learning method**.

We compare the mean computing time of all the feature extraction methods in Figure 4. First, we can see that DL-*l*_1_LS using active-set algorithm is much more efficient than DL-*l*_1_LS-IP, DL-*l*_1_LS-PX, and semi-NMF using multiplicative update rules. Second, the non-negative dictionary learning models are more efficient than the *l*_1_-regularized models. Therefore as in the sparse coding method, priority should be given to the non-negative models when attempting to use dictionary learning in an application.

## Conclusions

In this study, *l*_1_-regularized and non-negative sparse representation models are comprehensively studied for the classification of high-dimensional biological data. We give a Bayesian treatment to the models. We prove that the sparse coding and dictionary learning models are in fact equivalent to MAP estimations. We use different priors on the sparse coefficient vector and the dictionary atoms, which lead to various sparse representation models. We propose parallel active-set algorithms to optimize the sparse coding models, and propose a generic framework for dictionary learning. We reveal that the sparse representation models only use inner products of instances. Using this dimension-free property, we can easily extend these models to kernel versions. With the comparison with existing models for high-dimensional data, it is shown that our techniques are very efficient. Furthermore, our approaches obtained comparable or higher accuracies. In order to promote the research of sparse representation in bioinformatics, the MATLAB implementation of the sparse representation methods discussed in this paper can be downloaded at [27].

Our Bayesian treatment may inspire the readers to try other prior distributions in order to design new sparse representation models. It also helps to discover the similarity and difference between sparse representation and other dimension reduction techniques. Our kernel versions can also be used to classify tensor data where an observation is not a vector but a matrix or tensor [28]. They can also be applied in the biomedical text mining and interaction/relational data where only the similarities between instances are known.

We will apply our technique to other high-throughput data, such as microarray epigenomic data, gene copy number profiles, and sequence data. We will impose both sparse-inducing prior on dictionary atoms and coefficients. Inspired by Bayesian factor analysis, we will investigate the variable selection methods using sparse dictionary. The sparse dictionary analysis would help us to uncover the biological patterns hidden in the high-dimensional biological data. Furthermore, combining Bayesian sparse representation and Bayesian regression leads to Bayesian sparse representation regression model, which is very helpful for designing supervised dictionary learning. Finally we should mention that we are working on a decomposition method for sparse coding which is efficient on large-scale biological data where there are at least thousands of samples.

## Competing interests

The authors declare that they have no competing interests.

## Authors' contributions

YL proposed the original idea, did literature survey, implemented the methods, conducted the experiments, and wrote the first draft of this paper. AN supervised the whole research, gave constructive suggestions, and finished the final version of this paper.

## Declarations

The publication costs for this article were funded by Dr. Alioune Ngom with his Natural Sciences and Engineering Research Council of Canada (NSERC) Grants #RGPIN228117-2011.

This article has been published as part of *BMC Systems Biology *Volume 7 Supplement 4, 2013: Selected articles from the IEEE International Conference on Bioinformatics and Biomedicine 2012: Systems Biology. The full contents of the supplement are available online at http://www.biomedcentral.com/bmcsystbiol/supplements/7/S4.
